# Ketone body augmentation decreases methacholine hyperresponsiveness in mouse models of allergic asthma

**DOI:** 10.1016/j.jacig.2022.08.001

**Published:** 2022-09-07

**Authors:** Madeleine M. Mank, Leah F. Reed, V. Amanda Fastiggi, Paola E. Peña-García, Laura R. Hoyt, Katherine E. Van Der Vliet, Jennifer L. Ather, Matthew E. Poynter

**Affiliations:** Department of Medicine, Division of Pulmonary Disease and Critical Care, University of Vermont, and The Vermont Lung Center, Burlington.

**Keywords:** Asthma, ketogenic diet, ketones, methacholine, mice

## Abstract

**Background:**

Individuals with allergic asthma exhibit lung inflammation and remodeling accompanied by methacholine hyperresponsiveness manifesting in proximal airway narrowing and distal lung tissue collapsibility, and they can present with a range of mild-to-severe disease amenable or resistant to therapeutic intervention, respectively. There remains a need for alternatives or complements to existing treatments that could control the physiologic manifestations of allergic asthma.

**Objectives:**

Our aim was to examine the hypothesis that because ketone bodies elicit anti-inflammatory activity and are effective in mitigating the methacholine hyperresponsiveness associated with obese asthma, increasing systemic concentrations of ketone bodies would diminish pathologic outcomes in asthma-relevant cell types and in mouse models of allergic asthma.

**Methods:**

We explored the effects of ketone bodies on allergic asthma–relevant cell types (macrophages, airway epithelial cells, CD4 T cells, and bronchial smooth muscle cells) *in vitro* as well as *in vivo* by using preclinical models representative of several endotypes of allergic asthma to determine whether promotion of ketosis through feeding a ketogenic diet or providing a ketone precursor or a ketone ester dietary supplement could affect immune and inflammatory parameters as well as methacholine hyperresponsiveness.

**Results:**

In a dose-dependent manner, the ketone bodies acetoacetate and β-hydroxybutyrate (BHB) decreased proinflammatory cytokine secretion from mouse macrophages and airway epithelial cells, decreased house dust mite (HDM) extract–induced IL-8 secretion from human airway epithelial cells, and decreased cytokine production from polyclonally and HDM-activated T cells. Feeding a ketogenic diet, providing a ketone body precursor, or supplementing the diet with a ketone ester increased serum BHB concentrations and decreased methacholine hyperresponsiveness in several acute HDM sensitization and challenge models of allergic asthma. A ketogenic diet or ketone ester supplementation decreased methacholine hyperresponsiveness in an HDM rechallenge model of chronic allergic asthma. Ketone ester supplementation synergized with corticosteroid treatment to decrease methacholine hyperresponsiveness in an HDM-driven model of mixed-granulocytic severe asthma. HDM-induced morphologic changes in bronchial smooth muscle cells were inhibited in a dose-dependent manner by BHB, as was HDM protease activity.

**Conclusions:**

Increasing systemic BHB concentrations through dietary interventions could provide symptom relief for several endotypes of allergic asthmatic individuals through effects on multiple asthma-relevant cells.

## Graphical Abstract

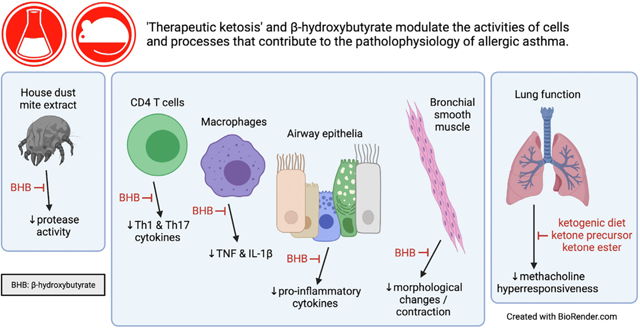

Asthma is a common, chronic pulmonary disorder that mechanistically involves a complex interaction of lung inflammation, remodeling, and reactivity.^1^ Allergic asthma increases responsiveness to bronchoconstricting agents, making both humans and mice hyperresponsive to the most common clinically used asthma diagnostic, methacholine.^2^ As can be effectively modeled in mice,^3^ allergic asthma typically manifests in large airway methacholine hyperresponsiveness and additionally involves distal lung compartments that are affected as a consequence of tethering to the airways.

Treatments for allergic asthma include bronchorelaxing β-agonists that increase airway lumen caliber, anti-inflammatory corticosteroids, and biologic therapies targeting causal mediators of the type2 immuneresponse.^4,5^ Althougheffective disease control is afforded to the majority of patients, there remain those with “difficult-to-treat” allergic asthma, for whom alternative or complementary therapies are needed.^6–8^ We recently reported on the beneficial effect of elevating circulating levels of ketone bodies, termed *therapeutic ketosis*, in mouse models of obese asthma,^9^ wherein they significantly decrease methacholine hyperresponsiveness. Ketone bodies can become elevated systemically as fatty acids consumed in the diet^10^ or mobilized from adipose tissue as a consequence of energetic demand^11–13^ are catabolized through β-oxidation in the liver to the ketone bodies acetoacetate (AcAc) and β-hydroxybutyrate (BHB), which are then released into the circulation and can be used as an energy source by cells throughout the body.^14^ Consuming a ketone body precursor such as 1,3-butanediol (1,3-BD)^15^ or ketone esters (KEs), a dietary supplement approved for human use,^16^ can transiently elevate ketone body concentrations.

Ketone bodies can modulate several of the key pathologic processes involved in both obese and allergic asthma.^16–18^ As an energy source, ketone bodies make cells less reliant on glycolysis,^14,19–23^ as a result of which they produce less lactic acid, a catabolite implicated as a causal factor in the pathogenesis of allergic asthma.^24–28^ Ketone bodies have been reported to function through cell surface receptors, including the G protein–coupled receptors hydroxycarboxylic acid receptor 2 (HCAR2/GPR109a) and free fatty acid receptor 3 (FFAR3/GPR41).^11,12,29,30^ Ketone bodies also function as antioxidants,^16,17,31,32^ and they exert anti-inflammatory effects, including suppression of nuclear factor-κB activation,^33^ as well as inhibition of the NLRP3 inflammasome and subsequent IL-1β production,^18,34–36^ which are also implicated in the pathogenesis of allergic asthma. Interestingly, IL-1β is itself a cause of elevated glycolysis and accompanying pathology in asthma.^25,37,38^ Alternate-day caloric restriction elevates BHB levels, which are correlated with reductions in oxidative stress and inflammation, along with improved clinical findings in overweight asthmatic subjects, including those with allergic asthma.^39^ Importantly, ketone body augmentation in human subjects is well tolerated.^40^

Despite the strong connections between the mechanisms underlying allergic asthma and the beneficial effects of ketone bodies, their potential to be used therapeutically in allergic asthma has not been evaluated in the modern era.^41,42^ We hypothesized that because ketone bodies can exert significant anti-inflammatory, redox-regulating, and metabolic effects, they could be relevant targets and tools in the treatment of allergic asthma. Our objectives were to evaluate the effectiveness of augmenting ketone body concentrations through dietary means on diminishing pathologic features of allergic asthma in several preclinical mouse models caused by exposure to the most common perennial allergen, house dust mites (HDMs) (*Dermatophagoides farina* and *Dermatophagoides pteronyssinus*),^43^ including those representing persistent and severe disease, as well as to identify mechanisms through which ketone bodies may modulate these effects. Further understanding the efficacy and mechanisms of ketone bodies *in vitro* and in preclinical mouse models *in vivo* could provide new dietary and pharmacologic targets for treatment of allergic asthma that could in turn be addressed in subsequent clinical trials.

## METHODS

### Study approval

The animal experiments were reviewed and approved by the University of Vermont’s Institutional Animal Care and Use Committee (protocols no. 18-023 and PROTO202000195), in accordance with the recommendations in the *Guide for the Care and Use of Laboratory Animals*, prepared by the Institute of Laboratory Animal Resources, National Research Council, and published by the National Academy Press (revised 2011). Studies involving potentially hazardous materials were reviewed and approved by the University of Vermont’s institutional biosafety committee (protocols no. 09-018 and REGF201900052).

### *In vitro* macrophage and airway epithelial cell studies

J774 murine macrophages purchased from the American Type Culture Collection (Manassas, Va) and murine bronchiolar epithelial cells (mouse SV40-transformed club cells)^44^ were cultured at37°C in 95% humidified air containing 5% CO_2_ by using Dulbecco modified Eagle medium (Thermo Fisher Scientific, Waltham, Mass) containing 10% FBS, 2 mM l-glutamine, 50 U/mL of penicillin, and 50 μg/mL of streptomycin (Life Technologies, Grand Island, NY).HBEC3-KT human bronchiolar epithelial cells obtainedfrom the American Type Culture Collection were cultured at 37°C in 95% humidified air containing 5% CO_2_ by using Dulbecco modified Eagle medium F-12 (Thermo Fisher Scientific) containing 10 ng/mL of cholera toxin, 10 ng/mL of epidermal growth factor, 5 μg/mLof insulin, 5 μg/mL of transferrin, 0.1 μM dexamethasone, 15 μg/mL of bovine pituitary extract, 0.5 mg/mL of BSA, 50 U/mL of penicillin, and 50 μg/mL of streptomycin (Life Technologies). For the experiments, J774 and mouse SV40-transformed club cells were plated at 1 × 10^6^ cells/mL in 125 μL of mediumin96-wellplates, whereas HBEC3-KT cells were plated at 2 × 10^5^ cells/mL in 500 μL of medium in 24-well plates. All cells were allowed to grow overnight. The following day, the medium was removed, fresh medium was added, and cells were treated as indicated within the figure legends for each experiment by using ultrapure *Escherichia coli* O111:B4 LPS and ATP (Invivogen, San Diego, Calif) in the absence or presence of AcAc, acetone, or BHB (Sigma-Aldrich, St. Louis, Mo). Cell supernatants were harvested at the end of each experiment, spun down at 3300 *g* for 10 minutes to pellet cellular debris, transferred to new tubes, and frozen at −20°C until analysis.

### *In vitro* T lymphocyte studies

To enrich naive CD4^+^ T cells, spleens and peripheral (inguinal and axial) lymph nodes of naive mice were dissociated through a 70-μm mesh filter (BD Biosciences, San Jose, Calif), and lymphocytes were enriched by centrifugation through the lymphocyte separation medium (MP Biomedicals, Irvine, Calif). CD4^+^ T cells were isolated via magnetic negative selection followed by depletion of CD25^+^ cells by positive selection using the manufacturer’s instructions (STEMCELL Technologies, Vancouver, Canada). Cells were resuspended in complete medium (RPMI-1640 supplemented with 5% FBS [Cell Generation, Fort Collins, Colo], 2500 μg/mL of glucose, 2 mM l-glutamine, 10 μg/mL of folic acid, 1 mM sodium pyruvate, 50 μM 2-mercaptoethanol, 100 U/mL of penicillin, and 100 μg/mL of streptomycin) containing 2 μg/mL of anti-CD28 (BD Pharmingen, San Diego, Calif) in wells of plates coated with anti-CD3 (BD Pharmingen) at a concentrationof 5 μg/mL. Naive cells from a portion of the aforementioned preparations were incubated at 2 × 10^6^ cells/mL in the absence (H_2_O vehicle) or presence of 10 mM AcAc, BHB, or 1,3-BD for 72 hours, after which conditioned medium was collected. Alternatively, a portion of the naive CD4^+^ T cells from the aforementioned preparations were polarized *in vitro*, as follows. For T_H_2 polarization, 30 ng/mL of IL-4, 20 IU/mL of IL-2, and 10 μg/mL of anti–IFN-*γ* were added. For T_H_17 polarization, 30 ng/mL of IL-6, 1 ng/mL of TGF-β, 10 ng/mL of IL-23, 10 μg/mL of anti–IFN-*γ*, and 10 μg/mL of anti–IL-4 were added. The cytokines and antibodies were from BD Pharmingen. On day 3, the cells were split into new anti-CD3–coated wells and fresh medium containing cytokines and/or antibodies were added. Cells were harvested on day 6, counted, and plated at 2 × 10^6^ cells/mL in complete medium in anti-CD3–coated wells in the absence (H_2_O vehicle) or presence of 10 mM AcAc, BHB, or 1,3-BD for 72 hours.

To enrich antigen-specific T cells, pooled mediastinal lymph nodes and spleens from mice subjected to an intranasal HDM sensitization and challenge model of eosinophilic allergic asthma were dissociated through a 70-μm mesh filter (BD Biosciences), and lymphocytes were enriched by centrifugation through lymphocyte separation medium (MP Biomedicals). Cells were counted with a hemocytometer, and 4 × 10^6^ cells/mL were cultured in RPMI-1640 supplemented with 5% FBS (Cell Generation), 2500 μg/mL of glucose, 2 mM l-glutamine, 10 μg/mL of folic acid, 1 mM sodium pyruvate, 50 μM 2-mercaptoethanol, 100 U/mL of penicillin, and 100 μg/mL of streptomycin and then restimulated in an antigen-specific manner with 15 μg/mL (by protein) of *Dermatophagoides pteronyssinus* HDM extract in saline (part no. XPB70D3A25, lot no. 343205 [Stallergenes Greer, Lenoir, NC]) in the absence or presence of increasing concentrations of AcAc or BHB. Supernatants were collected after 96 hours of incubation at 37°C in 5% CO_2_.

### *In vitro* bronchial smooth muscle cell studies

Human bronchial smooth muscle cells (HBSMCs) purchased from Lonza (Morristown, NJ) were cultured using the smooth muscle cell growth medium-02 BulletKit according to the manufacturer’s instructions at 37°C in 95% humidified air containing 5% CO_2_. The cells were used within the first 9 passages to ensure proper smooth muscle phenotype. For experiments, HBSMCs were plated at 5 × 10^4^ cells/mL in 500 μL of medium in 24-well plates and allowed to grow for 2 days. The medium was then removed, fresh medium was added, and cells were treated as indicated within the figure legends for each experiment by using HDM extract in the absence or presence of BHB for 24 hours. HBSMCs were imaged using a Nikon Eclipse TS100 microscope (Melville, NY) at ×10 magnification and equipped with a MU1003 AmScope Microscope Digital Camera (Irvine, Calif). For quantitative analysis, systemic uniform sampling by independent observers was used to select imaging locations. Images were binarized by using ImageJ technology (FIJI),^45^ and background was eliminated by using the raw images in the Image calculator function. Cellular surface area was quantified using the bwarea function in MATLAB, version R2022a (The Mathworks Inc, Natick, Mass). All experimental conditions were replicated.

### Protease activity assay

HDM-associated protease activity was measured by using a microplate assay in which HDM extract was incubated in the presence of 10 μg/mL of dye/quencher-ovalbumin (catalog no. D-12053, Molecular Probes, Eugene, Ore) without or with BHB at 37°C for 1 hour. Fluorescence intensity (excitation at 485 ± 20 nm and emission at 528 ± 20 nm) induced by the protease-dependent liberation of the quencher from the BODIPY FL fluorescent dye was read every minute on a Bio-Tek Synergy HTX multimode plate reader. End values are presented.

### Mice and diets

Six-week-old female C57BL/6J (stock no. 000664) or BALB/cJ (stock no. 000651) mice were purchased from The Jackson Laboratory (Bar Harbor, Me) and were allowed to acclimate in an American Association for the Accreditation of Laboratory Animal Care–accredited facility at the University of Vermont for at least 1 week before study initiation. The mice were maintained on a 12-hour light/dark cycle beginning at 0700 and 1900, respectively, and provided with irradiated chow (Prolab RMH 3000, catalog no. 3005984-712, LabDiet, St Louis, Mo) and autoclaved drinking water *ad libitum*. Chow containing 20% KE (wt/vol) (HVMN Inc, San Francisco, Calif) or 1,3-BD (Sigma-Aldrich) was prepared by pulverizing the chow with a food processor, incorporating the KE or 1,3-BD by using a kitchen stand mixer, forming the slurry into cubes using silicone ice cube trays in a freezer, and storing the cubes at −20°C until use. The KE contains 25 g of pure ketones per 65 mL and less than 2% stevia leaf extract, flavorings, and preservatives. Ketogenic diet (catalog no. D03022101) was purchased from Research Diets (New Brunswick, NJ) and stored frozen. The food in cages was replaced twice each week. Body weights were measured using a laboratory balance. Mice were humanely killed with sodium pentobarbital (150 mg/kg by intraperitoneal injection [Midwest Veterinary Supply, Lakeville, Minn]).

### Mouse models of eosinophilic asthma

For the alum and HDM (alum/HDM) model, mice were sensitized by administering 100 μL of an emulsification containing 50 μL of alum (Imject Alum, Pierce Biotechnology, Rockford, Ill) and 25 μg (by protein) of *Dermatophagoides pteronyssinus* HDM extract in saline (part no. XPB70D3A25, lot no. 343205 [Stallergenes]) via intraperitoneal injection on days 1 and 15. For the intranasal HDM model, isoflurane-anesthetized mice were sensitized by intranasal instillation of 10 μg (by protein) of HDM extract in 40 μL of saline on 2 occasions, as indicated in the figures and figure legends. For both models, antigen challenges (and rechallenges) were performed by intranasal inhalation of 10 μg of HDM in 40 μL of saline, as indicated in the figures and figure legends, and the mice were analyzed 1 day later.

### Mouse model of mixed-granulocytic severe asthma

For the complete Freund adjuvant (CFA) and HDM (CFA/HDM) model, mice were anesthetized with inhaled isoflurane and injected under the skin of their back with 100 μL of an emulsification containing 50 μL of CFA (Sigma-Aldrich) supplemented with 4 mg/mL of *Mycobacterium tuberculosis* extract (BD Biosciences) and 25 μg (based on protein content) of HDM extract in saline on day 1. Antigen challenges were performed by intranasal inhalation of 10 μg HDM in 40 μL of saline on days 19, 20, 21, and 22. Some mice were administered saline vehicle or treated with 2.5 mg/kg of dexamethasone (AgriLabs, St. Joseph, Mo) by intraperitoneal injection on days 19 and 21, immediately preceding the intranasal HDM challenge. The mice were analyzed on day 23.

### Assessment of pulmonary responsiveness to methacholine

Responsiveness to inhaled methacholine was assessed in closed-chest mice. The mice were anesthetized with intraperitoneally administered sodium pentobarbital (90 mg/kg), their trachea was cannulated with a blunted 18-g needle, and they were connected to a flexiVent computer-controlled small animal ventilator (SCIREQ, Inc, Montreal, Canada). The mice were ventilated at 200 breaths per minute with a 0.25-mL tidal volume and 3 cm H_2_O positive end-expiratory pressure (PEEP). Next, the mice were paralyzed with an intraperitoneal injection of pancuronium bromide (0.8 μg/kg). The mice were stabilized over about 10 minutes of regular ventilation at a positive PEEP of 3 cm H_2_O. A standard lung volume history was then established by delivering 2 total lung capacity maneuvers to a pressure limit of 25 cm H_2_O and holding for 3 seconds. Next, 2 baseline measurements of respiratory input impedance (Zrs) were obtained from 2-second multifrequency oscillations at a PEEP of 3 cm H_2_O. This was followed by an inhalation of aerosolized PBS (control) for 10 seconds, achieved by an in-line piezo electric nebulizer (Aeroneb, Aerogen, Galway, Ireland). Zrs was then measured every 10 seconds for 3 minutes (18 measurements of Zrs in total). This complete sequence of maneuvers and measurements was then repeated for aerosol exposures to saline and 3 ascending doses of aerosolized methacholine (12.5, 25, and 50 mg/mL). The data were fit to the constant phase model of the lung^46^ to provide values reflecting airway resistance, tissue damping, and tissue elastance. Individual data points were excluded when the coefficient of determination to the constant phase model was less than 0.85 or when the values were less than the baseline levels. All data points for a particular dose of methacholine in an individual mouse were excluded when 50% or more of the individual data points (>9 of 18) were excluded. The mean values (6 SEM) of airway resistance, tissue damping, and tissue elastance in each of the mouse groups at each incremental methacholine dose are reported.

### BALF collection, analysis, and lung processing

In some studies, following pulmonary function assessment, anesthetized mice, were lavaged through an 18-g tracheal cannula with 1 mL of room temperature Dulbecco PBS (Sigma-Aldrich). Cells were manually counted immediately in white blood cell stain (0.2 mg/mL of crystal violet in 2% acetic acid) by using a hemocytometer. The bronchoalveolar lavage fluid (BALF) was centrifuged at 400 *g* for 10 minutes at room temperature, and cell-free supernatants were frozen at −80°C until analysis of total protein content by bicinchoninic acid assay (Pierce Biotechnology). Cell pellets were resuspended in saline and mounted on slides by cytospin (100,000 cells per slide) for hematoxylin and eosin staining and differential analysis. Following collection of the BALF, the lungs were dissected, ground to a fine powder using a liquid nitrogen–chilled mortar and pestle, and stored at −80°C until analysis.

### Serum collection and BHB analysis

After the mice had been humanely killed at the completion of flexiVent analysis, approximately 300 μL of blood was collected via cardiac puncture from the right ventricle by using a 25-g needle attached to a 1-mL syringe. The blood was transferred into serum separator tubes (Becton Dickinson, Franklin Lanes, NJ) and centrifuged, and the serum was kept frozen at −80°C. The serum was used to determine the concentration of BHB (BHB colorimetric assay kit, Cayman Chemical, Ann Arbor, Mich) and immunoglobulins.

### Immunoglobulin immunoassays

For determination of total serum IgE by 2-step sandwich (capture) ELISA, 96-well plates were coated with 2 μg/mL of anti-mouse IgE mAb (BD Pharmingen clone R35–72) in PBS for 2 hours at 37°C. The plates were washed, blocked for 1 hour in PBS/1% BSA, and washed again, after which serum samples or IgE standard (BD Pharmingen, catalog no. 557079) were added in duplicate in PBS/1% BSA overnight at 4°C. For determination of HDM-specific IgG1 and IgG2c by indirect ELISA, 96-well plates were coated overnight at 4°C with 2 μg/mL of HDM extract in PBS (pH 7.2–7.4), washed with 0.05% Tween 20 in PBS, and blocked for 2 hours at room temperature with PBS/1% BSA. The plates were washed, and serum diluted in blocking solution was applied to the wells in triplicate over a series of eight 4-fold dilutions starting at 1:20 and incubated overnight at 4°C. For all isotypes, plates were washed after incubation with samples, and 2 μg/mL of biotinylated isotype-specific secondary antibodies (BD Biosciences) in 1% BSA/PBS were incubated in the plates at room temperature for 2 hours (for IgG2c, a cross-reactive antibody that recognizes IgG2a was used^47^). Plates were washed, and streptavidin-peroxidase (R&D Systems, Minneapolis, Minn) was incubated in the plates at room temperature for 30 minutes. Plates were washed and developed using reagents from R&D Systems; the reaction was stopped with 1N H_2_SO_4_, and ODs were read by using a Bio-Tek Instruments (Winooski, Vt) Synergy HTX multimode plate reader at 450 nm with background subtraction at 570 nm.

### Cytokine immunoassays

Conditioned medium from the cell culture studies was collected at the indicated time points, centrifuged to eliminate debris, transferred into new tubes or multiwell plates, and frozen at −20°C until analysis. The ELISAs to quantitate mouse CCL20, CXCL1, CXCL2, G-CSF, GM-CSF, IFN-*γ*, IL-1β, IL-4, IL-5, IL-6, IL-10, IL-13, IL-17A, RANTES, and TNF levels, as well as human IL-8/CXCL8 levels, were DuoSets from R&D Systems; they were used according to the manufacturer recommendations, with samples diluted to coincide with the range of the standards.

### Western blotting

Lung tissue samples were pulverized using liquid nitrogen–chilled mortars and pestles, after which small quantities of the powder was mixed with RIPA sample buffer (50 mM Tris pH 8, 150 mM NaCl, 1% Nonidet P-40, 0.5% deoxycholate, 0.1% SDS containing 1 mM sodium orthovanadate [Sigma-Aldrich], 1× protease mixture inhibitor [Sigma-Aldrich], and 1× PMSF [Sigma-Aldrich]) and incubated for 30 minutes on ice with occasional vortexing. Samples were centrifuged for 10 minutes at 16,000 *g* through Qiashredder tubes (Qiagen, Germany); the cleared lysates were transferred to new tubes, and the protein was quantitated by using a bicinchoninic acid assay (Pierce). Next, 30 μg of protein was mixed with Laemmli sample buffer containing 2-ME and incubated for 5 minutes at 100°C. Samples were cooled on ice, loaded onto SDS-PAGE gels, run in Tris-glycine-SDS running buffer, and transferred to nitrocellulose (β-actin) or polyvinylidene difluoride (PAD4) membranes. The blots were blocked in 5% nonfat milk/ris-buffered saline with Tween 20 for 2 hours at room temperature and placed in primary Ab (anti–β-actin from Sigma-Aldrich or anti-PAD4 from Abcam, Cambridge, United Kingdom) overnight at 4°C. The blots were then washed 3 times in Tris-buffered saline with Tween 20 for 10 minutes and placed in biotin-conjugated secondary antibody (GE Healthcare, Buckinghamshire, United Kingdom, or Jackson Immunore-search, West Grove, Pa) for 2 hours at room temperature. The blots were again washed and incubated in enhanced chemiluminescence reagent or Pico enhanced chemiluminescence reagent (Thermo Fisher Scientific) for 5 minutes before exposure to a digital imager to capture and quantitate images.

### Data acquisition, data availability, and statistical analysis

All of the experiments involved multiple mice per group and were replicated. The data sets generated during and/or analyzed during the current study are available from the corresponding author on reasonable request. Data were analyzed by 2-tailed unpaired *t* test or 1-way or 2-way ANOVA and Tu-key (when comparing each mean with every other mean) or Dunnett (when comparing each mean with a control mean) multiple comparisons *post hoc* testing using GraphPad Prism 9.1.2 for Windows (GraphPad Software, Inc., La Jolla, Calif; RRID:SCR_002798). Data are presented as means plus or minus SEMs from a representative experiment. *P* values less than 0.05 in the *t* test or the multiple comparisons *post hoc* test were considered statistically significant. Significance levels of the tested comparisons are indicated in the figure legends.

## RESULTS

### Macrophage and airway epithelial cell proinflammatory cytokine secretion are inhibited by the ketone bodies AcAc and BHB

**FIG 1.**
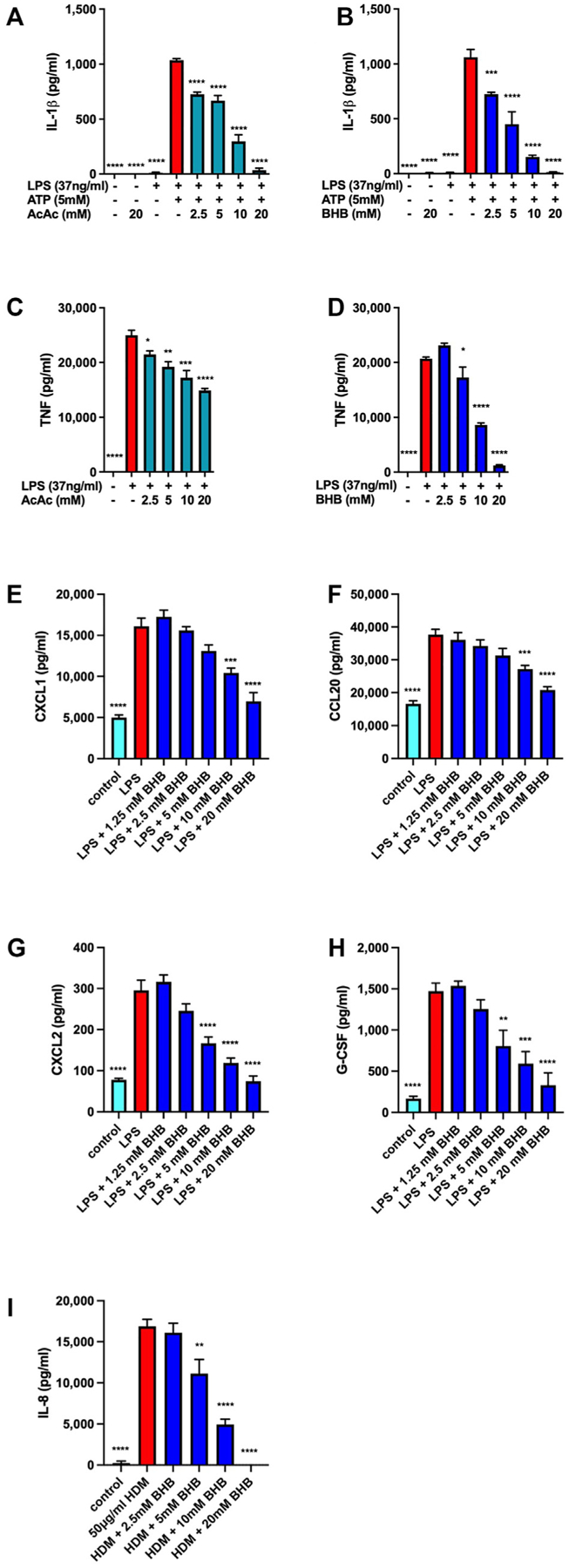
Ketone bodies decrease proinflammatory cytokine secretion from activated macrophages and airway epithelial cells. J774 macrophages were unstimulated or primed with LPS for 6 hours and then stimulated with ATP in the presence of increasing concentrations of AcAc **(A)** or BHB **(B)** for 1 hour and IL-1β level was measured. J774 macrophages were unstimulated (control) or stimulated with LPS in the presence of increasing concentrations of AcAc **(C)** or BHB **(D)** for 6 hours, and TNF level was measured. Mouse transformed airway epithelial club cells were unstimulated (control) or stimulated with LPS in the presence of increasing concentrations of BHB for 24 hours and CXCL1 **(E)**, CCL20 **(F)**, CXCL2 **(G)**, and G-CSF **(H)** levels were measured. Human airway epithelial club cells were unstimulated (control) or stimulated with HDM in the presence of increasing concentrations of BHB for 24 hours and IL-8/CXCL8 level was measured. Four samples per group. *P ≤ .05; **P ≤ .01; ***P ≤ .001; and **** *P* ≤ .0001 compared with LPS 1 ATP (**A** and **B**), LPS (**C-H**), or HDM (**I**).

Ketone bodies, especially AcAc and BHB, have been reported to exert anti-inflammatory effects, especially via inhibition of NLRP3 inflammasome activation.^18,34–36^ Using LPS-primed J774 mouse macrophages that we have reported to be susceptible to NLRP3 inflammasome inhibition by a number of short-chain alcohols,^48^ we confirmed that when AcAc (Fig 1, A) or BHB (Fig 1, B) was added immediately preceding ATP-induced NLRP3 inflammasome activation there was a dose-dependent decrease in the abundance of IL-1β that accumulated in the culture media. In addition, when added at the time of LPS-induced macrophage priming, both AcAc (Fig 1, C) and BHB (Fig 1, D) decreased TNF secretion; however, the inhibitory effects of BHB were more pro-found, especially at the 10-mM and 20-mM concentrations. Even at concentrations up to 20 mM, the third endogenous ketone body, acetone, did not inhibit ATP-induced TNF production or LPS plus ATP–induced IL-1β secretion (data not shown). Mouse airway epithelial club cells also exhibited dose-dependent decreases in the LPS-induced secretion of CXCL1, CCL20, CXCL2, and G-GSF when stimulated in the presence of increasing concentrations of BHB (Fig 1, E–H). Furthermore, exposure of HBEC3-KT transformed human bronchial epithelial cells (club cells) to the major asthma-associated perennial allergen, HDM extract, induced the production of IL-8 (CXCL8) that was inhibited by BHB in a dose-dependent manner.

### T lymphocyte cytokine production is inhibited by BHB

**FIG 2.**
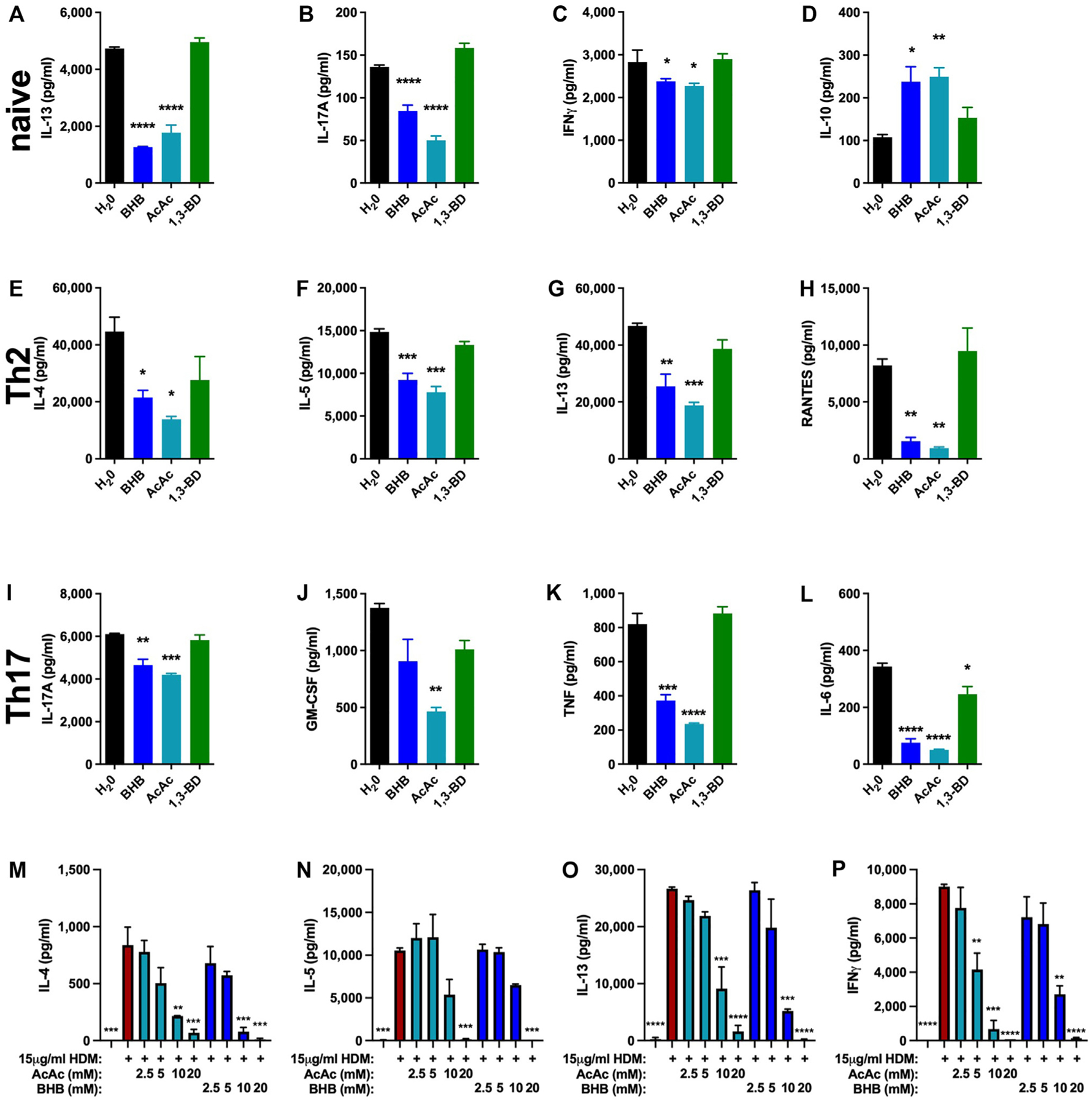
Ketone bodies decrease cytokine production from activated T lymphocytes. CD4^+^ T cells enriched from naive C57BL/6J mice cultured *in vitro* under naive **(A-D)**, T_H_2-**(E-H)**, or T_H_17-polarizing conditions **(I-L)** were restimulated with anti-CD3 without or with 10 mM BHB, AcAc, or 1,3-BD for 48 hours, and cytokine levels were measured from conditioned medium. Single-cell suspensions comprising splenocytes and mediastinal lymph node cells from C57BL/6J mice intranasally sensitized and challenged with HDM were restimulated *in vitro* without or with HDM in the absence of or in increasing concentrations of AcAc or BHB for 48 hours, and cytokines levels were measured from conditioned medium **(M-P)**. Four mice per group. *P ≤ .05; **P ≤ .01; ***P ≤ .001; and ****P ≤ .0001 compared with H_2_O (**A-L**) or HDM (**M-P**).

Expanding our *in vitro* studies to include additional cell types relevant to allergic asthma, we turned our focus to T lymphocytes. Naive CD4^+^ T cells stimulated with anti-CD3 plus anti-CD28 under control conditions (H_2_O vehicle) secreted IL-13, IL-17A, and IFN-*γ*, the levels of which were decreased when BHB or AcAc (but not the *in vivo* ketone body precursor 1,3-BD) were added during stimulation (Fig 2, A–C). Interestingly, production of IL-10, which is generally considered to be anti-inflammatory,^49^ was increased by BHB and AcAc relative to the control or 1,3-BD conditions (Fig 2, D). Naive CD4^+^ T cells that were polarized *in vitro* to T_H_2 and subsequently restimulated with anti-CD3 produced IL-4, IL-5, IL-13, and RANTES in levels that were decreased in the presence of 10 mM BHB or AcAc but not in the presence of 1,3-BD (Fig 2, E–H). Similarly, T_H_17-polarized CD4^+^ T cells restimulated in the presence of 10 mM BHB or AcAc secreted less IL-17A, GM-CSF, TNF, and IL-6 than did the control (H_2_O) cells or those stimulated in the presence of 1,3-BD (Fig 2, I–L). To examine the effect of ketone bodies on allergen-induced recall responses *ex vivo*, single-cell suspensions from lymph nodes and spleens of mice subjected to an intranasal HDM sensitization and challenge model of allergic asthma were restimulated *in vitro* with HDM in the presence of increasing concentrations of AcAc or BHB. Under control conditions, these cultures produced robust quantities of IL-4, IL-5, IL-13, and IFN-*γ*, which were decreased by AcAc and BHB in a dose-dependent manner (Fig 2, M–P).

### Methacholine hyperresponsiveness is decreased by feeding a ketogenic diet in an alum/HDM model of allergic asthma

**FIG 3.**
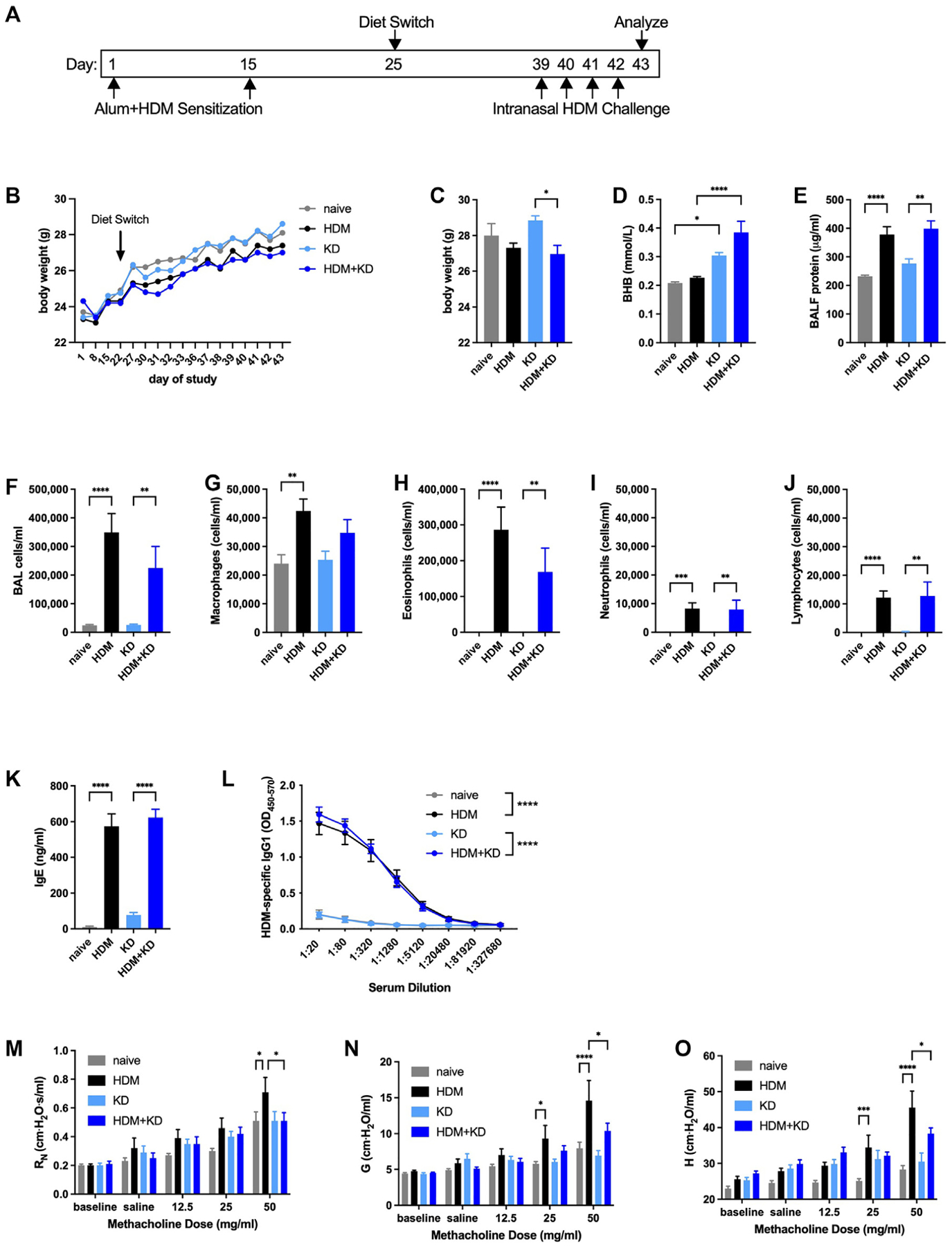
Feeding a ketogenic diet decreases methacholine hyperresponsiveness in an alum/HDM model of allergic asthma. C57BL/6J mice maintained with a diet of normal chow were kept naive or intraperitoneally sensitized with HDM absorbed to ImjectAlum (Alum) on days 1 and 15, maintained with a diet of normal chow, switched to a ketogenic diet (KD) beginning on day 25, and intranasally challenged daily with HDM on days 39 to 42. Mice were studied on day 43. Experimental time line **(A)** and body weights over the course of the study **(B)**. Body weights **(C)**, serum BHB level **(D)**, and BALF protein level **(E)** were measured at the end of the study. Total cell **(F)**, macrophage **(G)**, eosinophil **(H)**, neutrophil **(I)**, and lymphocyte **(J)** counts were measured from BALF. Total IgE **(K)** and HDM-specific IgG1 **(L)** levels were measured from serum. Airway resistance (R_N_) **(M)**, tissue damping (G) **(N)**, and tissue elastance (H) **(O)** were measured. Ten mice per group. *P ≤ .05; **P ≤ .01; ***P ≤ .001; and ****P ≤ .0001 compared with the indicated group.

Having observed substantial inhibitory effects of the major circulating ketone bodies BHB and AcAc in allergic asthma–relevant cell types, we sought to examine the effects of raising BHB concentrations on important outcomes in mouse allergic models *in vivo*. We began by using a model in which C57BL/6J mice were kept naive or were allergen-sensitized via intraperitoneal injection with HDM in the adjuvant Imject Alum and provided with normal chow or a ketogenic diet containing 80% of calories from fat, 20% of calories from protein, and essentially no carbohydrates, as we have used previously,^9^ beginning 10 days after the second allergen sensitization. Two weeks later, HDM-sensitized mice were intranasally challenged daily with 10 μg of HDM extract and studied 24 hours after the fourth challenge (Fig 3, A). As assessed by measuring all of the mice in each group (not individual mice), body weights followed similar trajectories over the course of the study, with the mice that had been sensitized and challenged with HDM displaying signs of decreased weight gain (Fig 3, B). At the end of the study, the mice that had been sensitized and challenged with HDM and fed a ketogenic diet weighed significantly less than the naive mice fed a ketogenic diet, although the differences were not substantial (Fig 3, C). Serum BHB concentrations were significantly increased in mice fed a ketogenic diet (Fig 3, D). In this alum/HDM model of allergic asthma, the level of BALF protein, a reflection of airway inflammation and damage, was increased compared with that in the naive control mice, and feeding the ketogenic diet had no effect on this outcome (Fig 3, E). Similarly, the counts of total HDM-induced BALF cells and the subtypes therein, predominantly eosinophils, were unaffected by ketogenic diet (Fig 3 F–J). Serum concentrations of total IgE (Fig 3, K) and HDM-specific titers of IgG1 (Fig 3, L) were strongly induced by alum/HDM sensitization and HDM challenge in mice fed normal chow, effects that were unaffected by ketogenic diet. Despite the apparent lack of anti-inflammatory or immunomodulatory effects of a ketogenic diet, the significant increases in HDM-induced methacholine hyperresponsiveness in the parameters of central airway (newtonian) resistance, tissue damping, and tissue elastance in mice fed normal chow were not present in mice fed a ketogenic diet (Fig 3, M–O).

### Methacholine hyperresponsiveness is decreased by feeding a ketogenic diet, providing a ketone body precursor, or providing a KE supplement in an intranasal HDM model of allergic asthma

**FIG 4.**
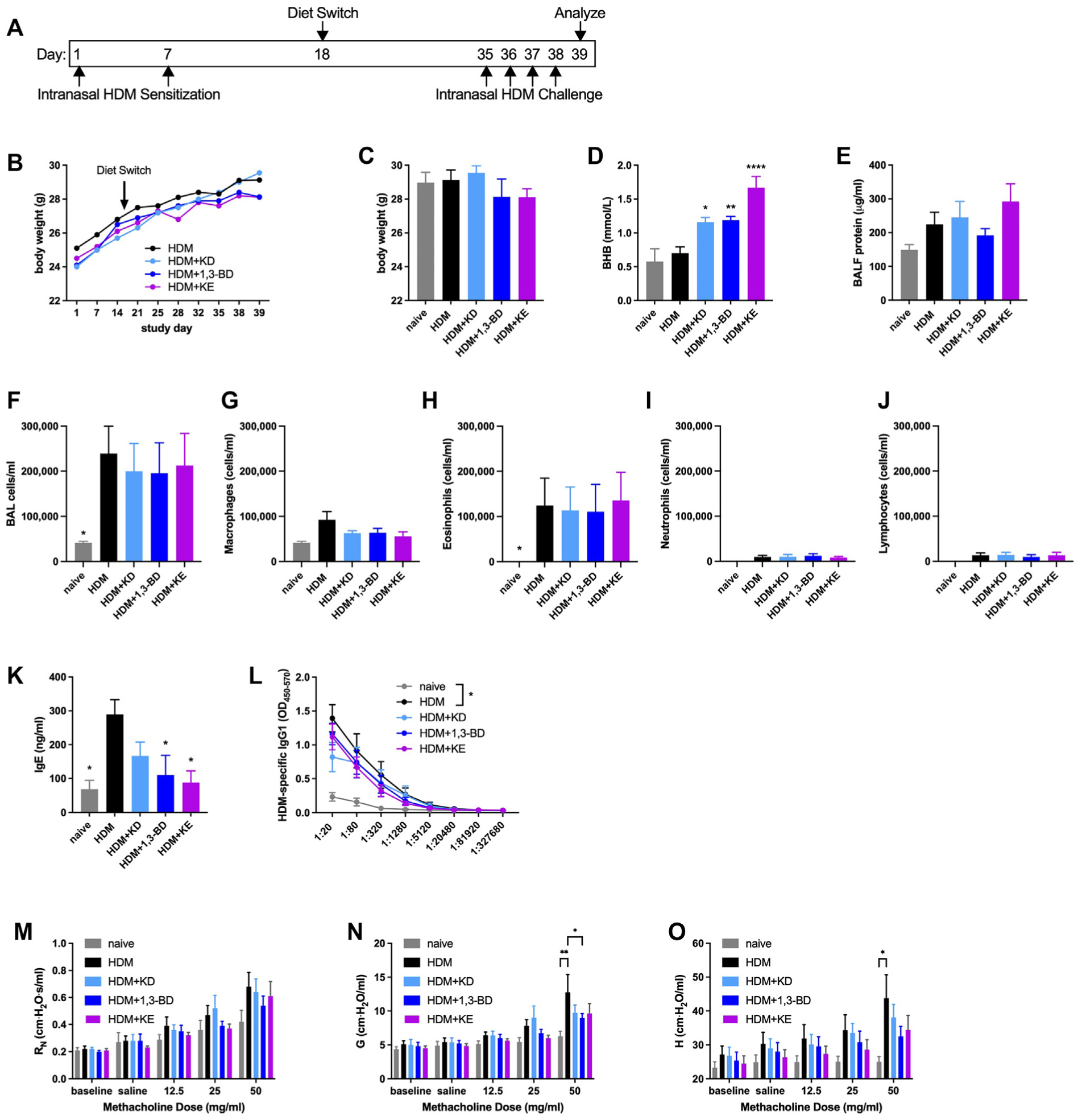
Ketogenic diet feeding, ketone body precursor supplementation, or KE supplementation decrease methacholine hyperresponsiveness in an intranasal HDM model of allergic asthma. C57BL/6J mice maintained on a diet of normal chow were kept naive or intranasally sensitized with HDM on days 1 and 7, maintained on a diet of normal chow, switched to a ketogenic diet (KD), switched to chow supplemented with 1,3-BD or switched to chow supplemented with KE beginning on day 18, and intranasally challenged daily with HDM on days 35 to 38. Mice were studied on day 39. Experimental time line **(A)** and body weights over the course of the study **(B)**. Body weights **(C)**, serum BHB level **(D)**, and BALF protein level **(E)** were measured at the end of the study. Total cell **(F)**, macrophage **(G)**, eosinophil **(H)**, neutrophil **(I)**, and lymphocyte **(J)** levels were measured from BALF. Total IgE **(K)** and HDM-specific IgG2c and HDM-specific IgG1 **(L)** were measured from serum. Airway resistance (R_N_) **(M)**, tissue damping (G) **(N)**, and tissue elastance (H) **(O)** were measured. Ten mice per group. *P ≤ .05; **P ≤ .01; ***P ≤ .001; and ****P ≤ .0001 compared with HDM (**C-K**) or the indicated group (**L-O**).

We next used a model in which C57BL/6J mice were kept naive or were allergen-sensitized via intranasal instillation with 10 μg of HDM extract (without additional adjuvant) on days 1 and 7 and provided with normal chow, a ketogenic diet, chow supplemented with 20% (by weight) of the BHB precursor 1,3-BD or 20% KE, as we used previously,^9^ beginning 11 days after the second allergen sensitization. Two weeks and 3 days later, HDM-sensitized mice were intranasally challenged daily with 10 μg of HDM extract and studied 24 hours after the fourth challenge (Fig 4, A). The body weights of the mice in each of the groups of mice (not individual mice) sensitized and challenged with HDM exhibited similar trajectories over the course of the study (Fig 4, B), with no differences in body weights between groups at the end of the study (Fig 4, C). Serum BHB concentrations were significantly increased in the mice fed a ketogenic diet or provided with supplementation with 1,3-BD or KE (Fig 4, D). In this milder model of allergen sensitization, the level of BALF protein was not significantly elevated compared with that obtained from naive mice or affected by diet (Fig 4, E). The total counts of BALF cells and the subtypes therein (predominantly comprising eosinophils) were significantly elevated in mice sensitized and challenged with HDM, but they were not affected by diet (Fig 4, F–J). The serum concentrations of total IgE were significantly decreased compared with those with HDM alone in mice supplemented with 1,3-BD or KE (Fig 4, K), but the significant elevations in HDM-specific of IgG1 titers in mice that had been sensitized to and challenged with HDM mice were unaffected by diet (Fig 4, L). Although central airway resistance was not significantly increased by intranasal HDM sensitization and challenge (Fig 4, M), significant increases in tissue damping and tissue elastance in mice fed normal chow were not present in mice fed a ketogenic diet or supplemented with 1,3-BD or KE (Fig 4, N–O).

### Methacholine hyperresponsiveness is decreased by KE supplementation in BALB/cJ mice subjected to an intranasal HDM model of allergic asthma

**FIG 5.**
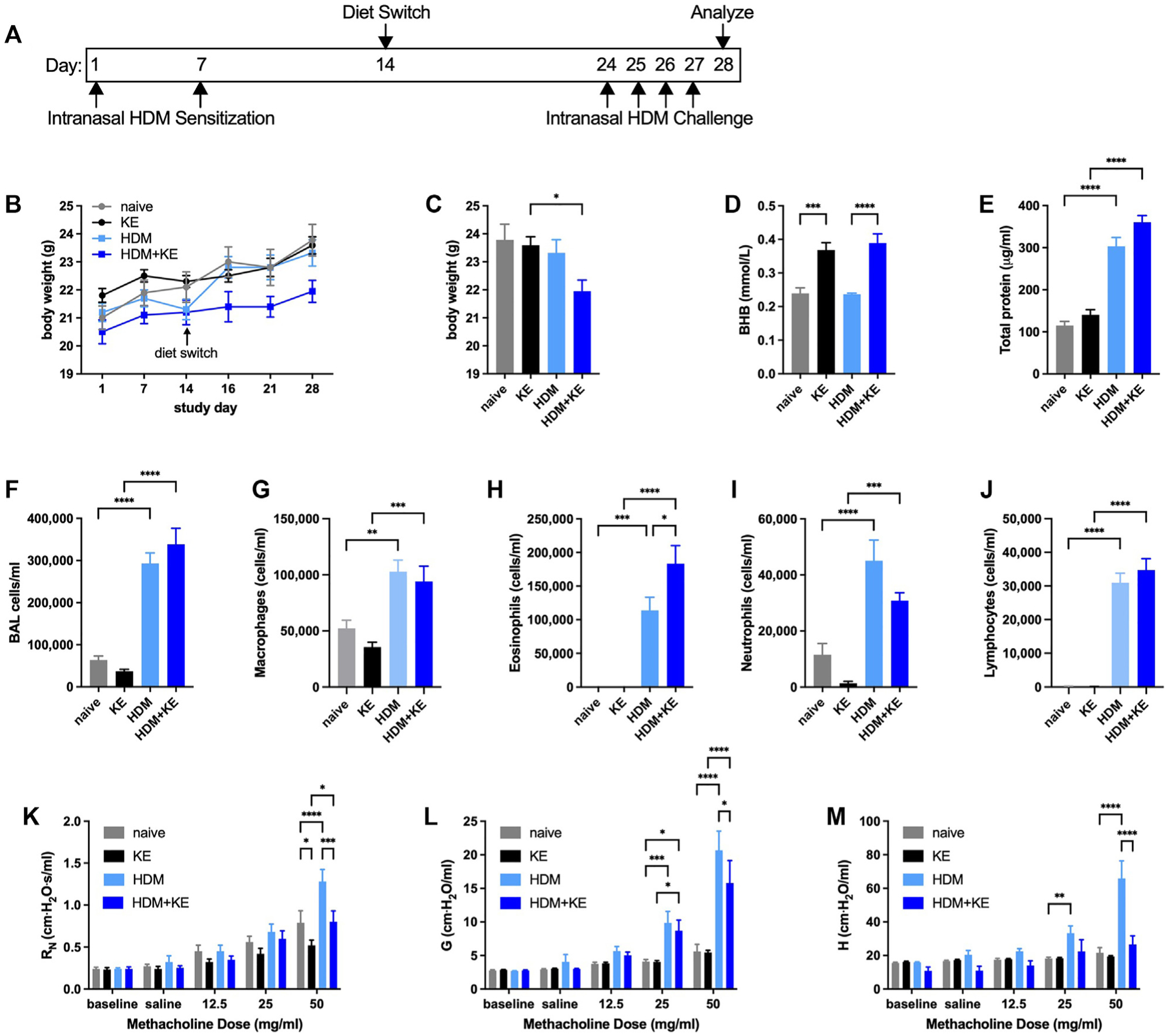
KE supplementation decreases methacholine hyperresponsiveness in an intranasal HDM model of allergic asthma in BALB/cJ mice. BALB/cJ mice maintained on a diet of normal chow were kept naive or intranasally sensitized with HDM on days 1 and 7, maintained on a diet of normal chow or switched to chow supplemented with KE beginning on day 14, and intranasally challenged daily with HDM on days 24 to 27. Mice were studied on day 28. Experimental time line **(A)** and body weights over the course of the study **(B)**. Body weights **(C)**, serum BHB levels **(D)**, and BALF protein levels **(E)** were measured at the end of the study. Total cell **(F)**, macrophage **(G)**, eosinophil **(H)**, neutrophil **(I)**, and lymphocyte **(J)** levels were measured from BALF. Airway resistance (R_N_) **(K)**, tissue damping (G) **(L)**, and tissue elastance (H) **(M)** were measured. Ten mice per group. *P* ≤ .05; **P ≤ .01; ***P ≤ .001; and ****P ≤ .0001 compared with the indicated group.

Because BALB/cJ mice are considered by many to be a model of inherent T_H_2 bias^50^ in which allergic responses are exaggerated, we next conducted a similar intranasal HDM sensitization and challenge study in this strain. Mice were either kept naive or allergen-sensitized on days 1 and 7 and provided with normal chow or chow supplemented with 20% KE beginning 1 week after the second allergen sensitization. Ten days later, the HDM-sensitized mice were intranasally challenged daily with 10 μg of HDM extract and studied 24 hours after the fourth challenge (Fig 5, A). The mice that had been sensitized with HDM and given KE displayed decreased body weight gain over the course of the study and were substantially lighter at the end of the study than the KE-fed mice not subjected to HDM sensitization and challenge (Fig 5, B and C). Whereas serum BHB concentrations were significantly increased in the mice provided with KE supplementation (Fig 5, D), the HDM-induced increases in BALF total protein (Fig 5, E) and BALF cellularity (Fig 5, F–J) were unaffected by KE supplementation. Nevertheless, HDM-induced methacholine hyperresponsiveness was significantly decreased in the mice supplemented with KE (Fig 5, K–M).

### Methacholine hyperresponsiveness is decreased by feeding a ketogenic diet or providing KE supplementation in an intranasal HDM rechallenge model of chronic allergic asthma

**FIG 6.**
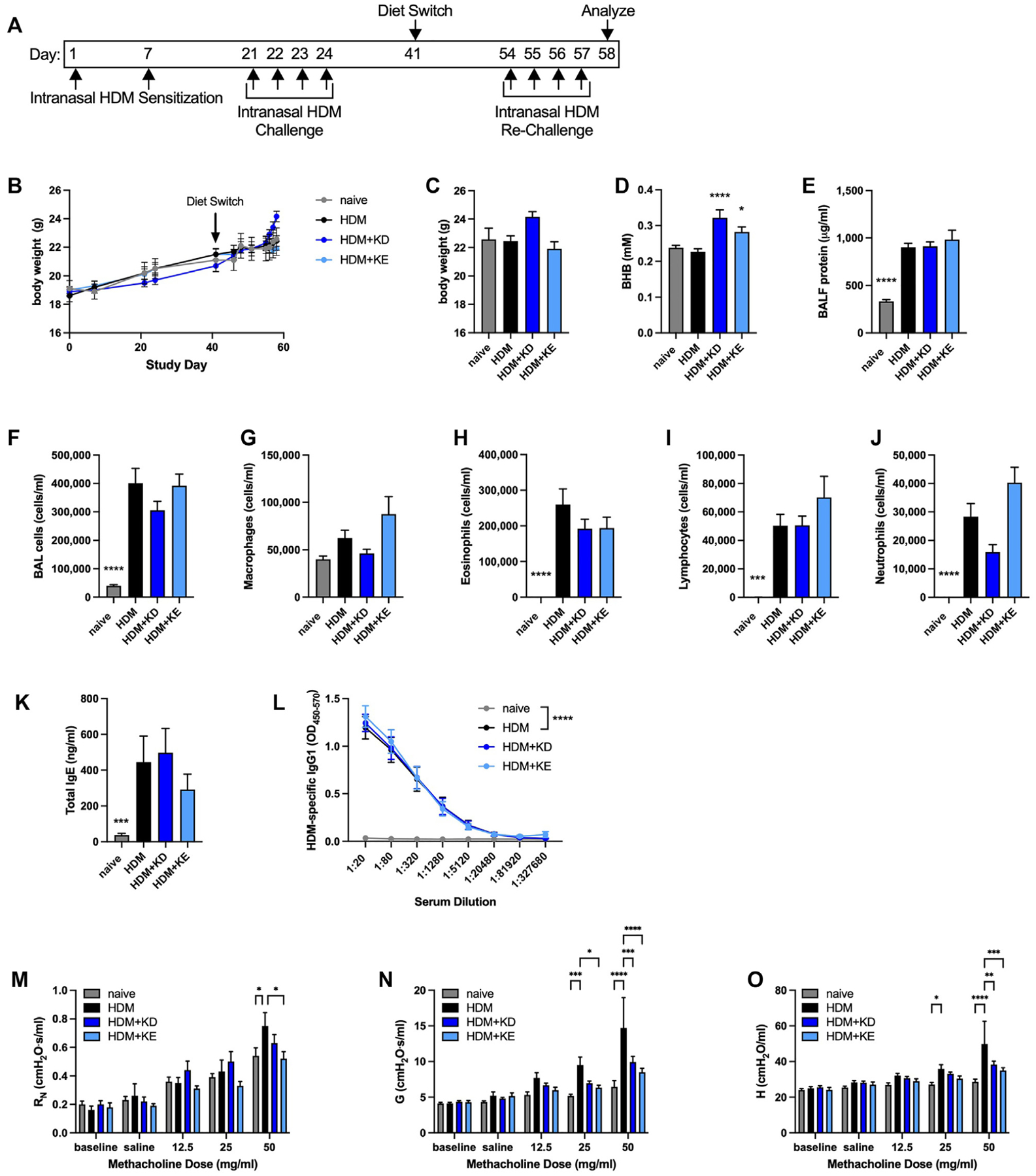
Ketogenic diet feeding or KE supplementation decrease methacholine hyperresponsiveness in an intranasal HDM rechallenge model of chronic allergic asthma. C57BL/6J mice maintained on a diet of normal chow were kept naive or intranasally sensitized with HDM on days 1 and 7, intranasally challenged daily with HDM on days 21 to 24, maintained on a diet of normal chow (NC), switched to a ketogenic diet (KD), or switched to chow supplemented with KE beginning on day 41, and intranasally challenged daily with HDM on days 54 to 57. Mice were studied on day 58. Experimental time line **(A)** and body weights over the course of the study **(B)**. Body weights **(C)**, serum BHB levels **(D)**, and BALF protein levels **(E)** were measured at the end of the study. Total cell **(F)**, macrophage **(G)**, eosinophil **(H)**, neutrophil **(I)**, and lymphocyte **(J)** levels were measured from BALF. Total IgE **(K)** and HDM-specific IgG1 **(L)** levels were measured from serum. Airway resistance (R_N_) **(M)**, tissue damping (G) **(N)**, and tissue elastance (H) **(O)** were measured. Ten mice per group. *P ≤ .05; **P ≤ .01; ***P ≤ .001; and ****P ≤ .0001 compared with HDM (**C-K**) or the indicated group (**L-O**).

We next sought to model a more persistent model of allergic asthma in which mice undergo allergen sensitization and challenge, accompanied by inflammatory influx and anatomic remodeling, and are then rechallenged with allergen^51^ in the absence or presence of dietary interventions to augment circulating BHB concentrations. For this model, C57BL/6J mice that had been provided with normal chow were kept naive or were intranasally HDM-sensitized on days 1 and 7 and then intranasally challenged daily with HDM extract on days 21 to 24. On day 41, the mice continued to receive normal chow or were switched to a ketogenic diet or chow supplemented with KE. On day 54, the mice that had been previously sensitized and challenged with allergen were intranasally HDM rechallenged daily and studied 24 hours after the fourth challenge (Fig 6, A). The body weights of the mice in each of the groups did not differ over the course of the study (Fig 6, B). Although there was a trend toward additional weight gain in the HDM-exposed mice being fed the ketogenic diet near the final days of the study, there were no significant differences in body weights between groups at the end of the study (Fig 6, C). Serum BHB concentrations were modestly, but significantly, increased in mice fed a ketogenic diet or provided with KE supplementation (Fig 6, D). The levels of BALF protein (Fig 6, E) and total BALF cells (Fig 6, F)—in particular eosinophils, lymphocytes, and neutrophils (Fig 6, G–J)—were significantly increased in the HDM-rechallenged mice but were not affected by diet. Similarly, the significant HDM-induced increases in serum total IgE and HDM-specific IgG1 were unaffected by diet (Fig 6, K and L). The values of all 3 parameters of methacholine responsiveness, namely, central airway resistance, tissue damping, and tissue elastance, were significantly increased in this model of intranasal HDM sensitization, challenge, and rechallenge (Fig 6, M–O), with decreased methacholine hyperresponsiveness in the mice fed a ketogenic diet (tissue damping and tissue elastance) and the mice supplemented with KE (airway resistance, tissue damping, and tissue elastance).

### KE supplementation and corticosteroid treatment synergize to decrease methacholine hyperresponsiveness in a model of antigen-driven mixed-granulocytic severe asthma

**FIG 7.**
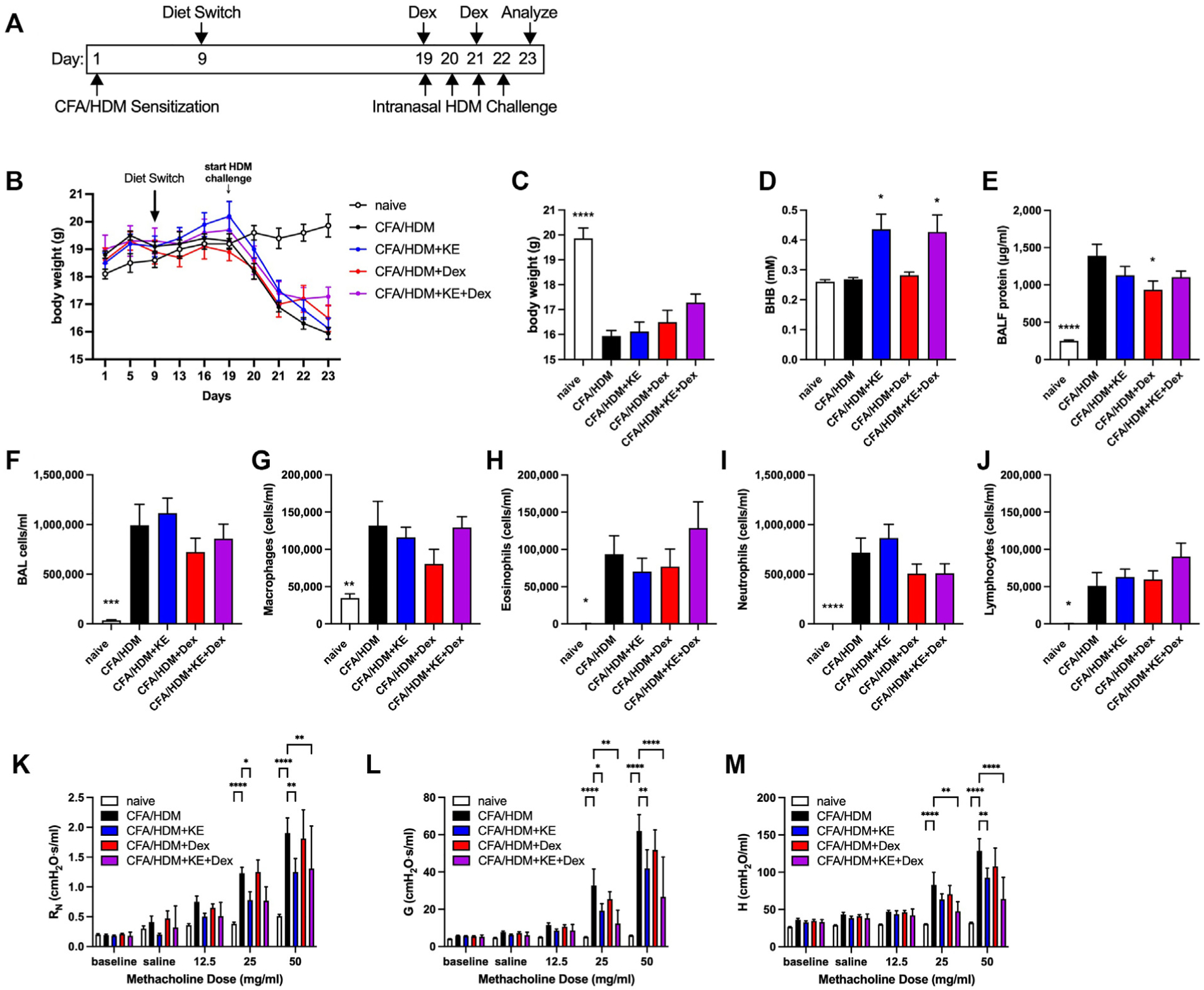
KE supplementation synergizes with corticosteroid treatment to decrease methacholine hyperresponsiveness in a model of antigen-driven mixed-granulocytic severe asthma. C57BL/6J mice maintained on a diet of normal chow were kept naive or subjected to the CFA/HDM model sensitization on day 0, maintained on a diet of normal chow or chow supplemented with KE beginning on day 9, and intranasally challenged daily with HDM on days 19 to 22 without or with the administration of 2.5 mg/kg of dexamethasone (Dex) on days 19 and 21 during the HDM challenge. Mice were studied on day 23. Experimental time line **(A)** and body weights over the course of the study **(B)**. Body weights **(C)**, serum BHB levels **(D)**, and BALF protein levels **(E)** were measured at the end of the study. Total cell **(F)**, macrophage **(G)**, eosinophil **(H)**, neutrophil **(I)**, and lymphocyte **(J)** levels were measured from BALF. Airway resistance (R_N_) **(K)**, tissue damping (G) **(L)**, and tissue elastance (H) **(M)** were measured. Ten mice per group. *P ≤ .05, **P ≤ .01, ***P ≤ .001, and ****P ≤ .0001 compared with CFA/HDM (**C-J**) or the indicated group (**K-M**).

Another criticism of the routinely used mouse allergic asthma models is that they are typically sensitive to the anti-inflammatory and immunosuppressive effects of corticosteroids, which are a mainstay of asthma therapy. However, those with severe asthma are resistant to the effects of systemic steroids and require additional therapeutics for asthma management.^6–8^ We recently reported a mouse model of severe asthma in which mixed granulocytic airway inflammation is accompanied by steroid resistance,^52^ which we used to evaluate the effectiveness of KE supplementation, alone or in combination with dexamethasone, to attenuate pathologic outcomes. For this model, C57BL/6J mice that had been provided with normal chow were kept naive or were sensitized subcutaneously with HDM emulsified in CFA on day 1. On day 9, the mice continued to receive normal chow or were switched to chow supplemented with KE. On day 19, the mice previously sensitized with CFA/HDM were intranasally HDM-challenged daily and studied 24 hours after the fourth challenge. On days 19 and 21 (immediately following the intranasal HDM challenge), 2 groups of mice were intraperitoneally administered 2.5 mg/kg of dexamethasone, a dose that elicits substantial therapeutic effects in other models of allergic asthma but not in this model of severe disease^52^ (Fig 7, A). The body weights of the mice in each of the groups did not differ until after the HDM challenge, at which time body weights fell precipitously in all of the mice subjected to the CFA/HDM model (Fig 7, B). At the end of the study, there was a significant decrease in the weight of the CFA/HDM mice, with no effect of the KE supplement, systemic corticosteroid treatment, or a combination thereof (Fig 7, C). Serum BHB concentrations were significantly increased in the mice provided with KE supplementation, and there was no interaction with dexamethasone (Fig 7, D). BALF protein levels were substantially and significantly elevated in the mice subjected to the CFA/HDM model, levels that were slightly, albeit significantly, decreased in the mice administered dexamethasone (Fig 7, E). BALF cellularity was very high and dominated by neutrophils, with no inhibition by KE supplementation, dexamethasone, or the combination (Fig 7, F–J). Serum total IgE and HDM-specific IgG1 levels were substantially elevated in the mice subjected to the CFA/HDM model and were unaffected by KE, dexamethasone, or a combination thereof (Fig E1, A and B in the Online Repository at www.jaci-global.org). The CFA/HDM model also induces robust levels of antigen-specific IgG2c (52), the levels of which were significantly decreased in mice administered dexamethasone without or with KE supplementation (Fig E1, C). Whereas lung tissue levels of IL-1β were significantly increased in the mice subjected to the CFA/HDM model and unaffected by KE or dexamethasone (Fig E1, D), CFA/HDM-induced increases in lung tissue IL-6 were reduced by dexamethasone but did not reach statistical significance in the group receiving dexamethasone plus KE (Fig E1, E). Neutrophils, neutrophil extracellular traps (NETs), and neutrophil ghosts are associated with severe asthma.^53,54^ NET formation requires activity of neutrophil PAD4 to enable histone citrullination preceding chromatin decondensation and NET expulsion.^55^ Citrullinated histone H3 concentrations are elevated in the systemic circulation of asthmatic individuals and are correlated with decreased lung function.^56^ As neutrophilic airway inflammation is a major component of the CFA/HDM model, we measured the abundance of PAD4 in the lungs. The CFA/HDM model substantially increased levels of PAD4 protein in lung lysates, and although small decreases in the mice provided with KE without or with dexamethasone were apparent, they did not reach statistical significance (Fig E1, F and G). The CFA/HDM model elicited substantial methacholine hyperresponsiveness. KE supplementation significantly decreased central airway resistance (Fig 7, K), whereas dexamethasone did not. Moreover, the inhibitory effects of KE on tissue damping (Fig 7, L) and tissue elastance (Fig 7, M) were even more confidently decreased in mice administered a combination of KE and dexamethasone, whereas the corticosteroid alone exerted no effect on any parameter of methacholine hyperresponsiveness.

### Bronchial smooth muscle contraction and HDM extract protease activity are inhibited by BHB

**FIG 8.**
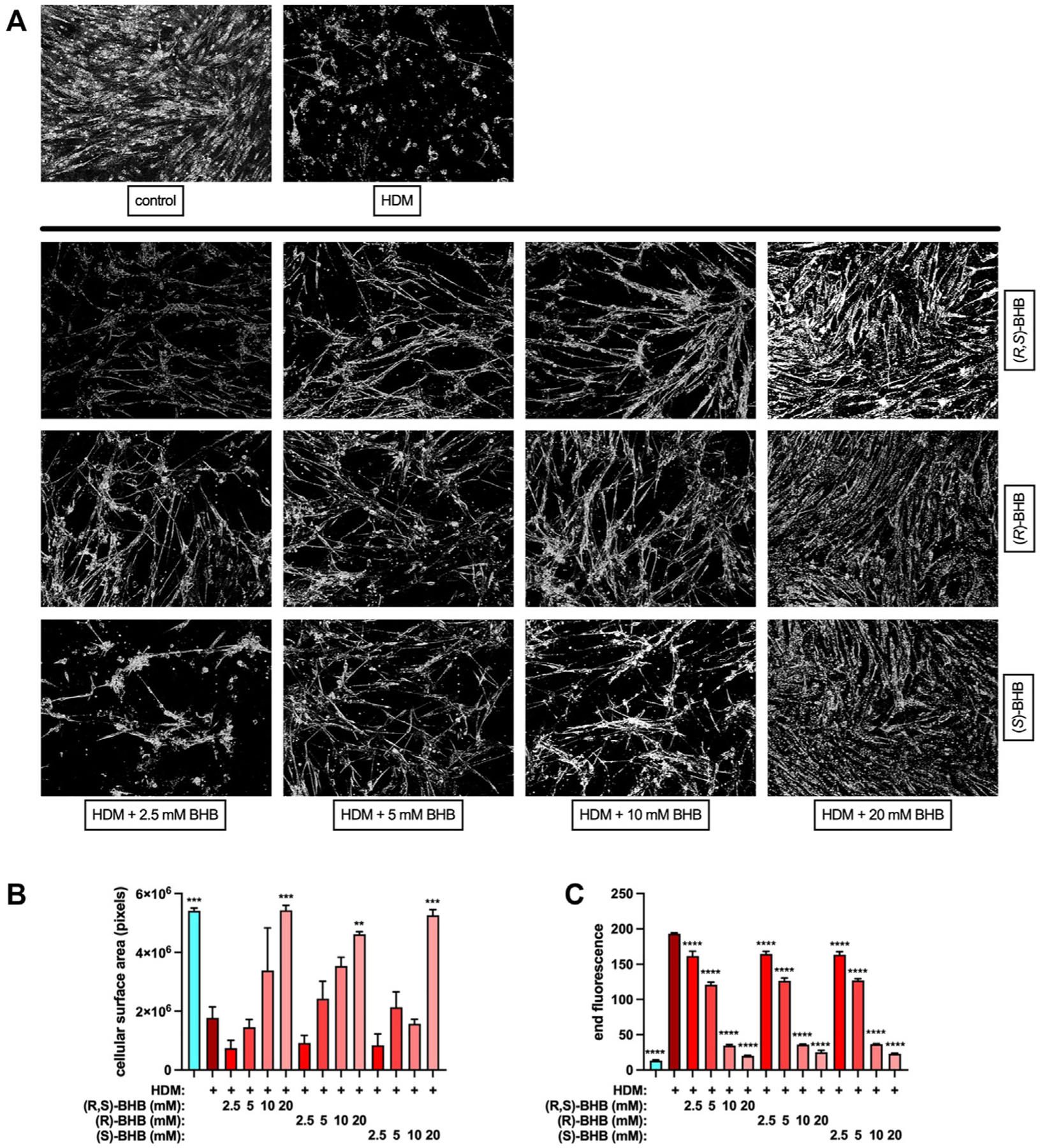
BHB decreases bronchial smooth muscle contraction and inhibits protease activity of HDM extract. HBSMCs cultured *in vitro* for 24 hours in the absence (control) or presence of HDM extract and increasing concentrations of a racemic BHB mixture or individual enantiomers of BHB were visualized by light microscopy for a contractive phenotype **(A)**. The pixel count of cells in the images were quantitated **(B)**. Images and values are representative of studies performed twice. HDM was incubated in the absence of or in increasing concentrations of BHB and a fluorogenic protease substrate for 1 hour and fluorescence was measured **(C)**. Four samples per group. ****P ≤ .0001 compared with HDM.

The results from the animal models of allergic asthma that are presented herein, as well as the mouse models of obesity-associated asthma reported previously,^9^ suggest prominent effects of ketone bodies on bronchial smooth muscle. Consequently, we exposed HBSMCs to HDM in the absence or presence of a BHB racemic mixture, (*R,S*)-BHB (used in Figs 1 and 2), as well as single (*R*)-BHB and (*S*)-BHB enantiomers, and visualized cell surface area by using light microscopy (Fig 8, A). In contrast to unexposed control cells, HBSMCs exposed to HDM displayed a contracted phenotype in which the cell area of the unexposed, confluent cells in culture were markedly condensed. The presence of BHB, whether the mixed enantiomer or single enantiomers, decreased HDM-induced HBSMC contraction. Quantitation of cell pixel density revealed that HBSMC contraction induced by HDM was inhibited in a dose-dependent manner irrespective of the BHB enantiomer (Fig 8, B). As allergen-associated proteases are an important means of HDM-induced cellular activation,^57^ and HDM protease can directly influence bronchial smooth muscle cell morphology,^58^ we examined the effect of several BHB enantiomers on HDM protease activity.^5^ This assay is sensitive to both selective serine (eg, trypsin) and cysteine (eg, papain) proteases, as well as to the mixture of serine and cysteine proteases present in HDM extract.^59^ The BHB racemic mixture and the single enantiomers equally inhibited HDM protease activity in a dose-dependent manner (Fig 8, C), implicating an absence of BHB stereoselectivity in this effect.

## DISCUSSION

The increasingly prevalent global asthma epidemic^60^—especially the epidemic of severe asthma^6–8^—has created a pressing need to devise alternative and complementary strategies to limit the impact on patients’ lives imposed by the syndrome. “Therapeutic ketosis” is one such approach that has recently gained attention for its potential to provide benefit in a myriad of disease settings and through a number of mechanisms. As reported herein, our studies demonstrate that the ketone bodies AcAc and BHB inhibit agonist-induced cytokine production from several asthma-relevant cell types, including proinflammatory cytokine production from macrophages and airway epithelial cells, secretion of prototypic cytokines from polyclonally stimulated or restimulated CD4^+^ T cells, and type 2 cytokines and IFN-*γ* secretion from HDM allergen–restimulated lymphocyte cultures. The mechanisms whereby these ketones affect a myriad of cell types *in vitro* remain uncertain, but they may include activation of cell surface receptors, providing energetic substrates for utilization through the Krebs cycle, functioning as antioxidants, modulating intracellular signaling regulating cytokine production and cell contraction, and inhibiting allergen protease activity. Moreover, through these or other mechanisms, dietary treatments that increase systemic concentrations of BHB *in vivo*, including a ketogenic diet, ketone body precursor feeding, or KE supplementation decrease allergic asthma–associated methacholine hyperresponsiveness, the most relevant pathophysiologic manifestation of preclinical asthma models.

Both large airway and peripheral lung dysfunction are present in allergic asthma,^61^ and our interventions show that increased BHB concentrations benefit both of these anatomic units. In each of the preclinical allergic asthma models presented, ketone bodies decreased the parameter airway resistance, which provides a measure of the flow resistance of the entire airway tree. Ketone body augmentation also decreased tissue damping and tissue elastance, which are increased by the development of heterogeneous ventilation to the distal reaches of the lung owing to variations in airway narrowing, and also by derecruitment of lung units,^62^ both of which are particularly sensitive to contraction of peripheral airways.^63^ As the central airways and the lung periphery are not mechanically independent owing to the tethering of parenchymal tissues to the airways,^64^ we speculate that a mechanism through which ketones restrain airway hyperresponsiveness may involve affecting bronchial smooth muscle cells affected by HDM challenges, attenuating their capacity to contract in response to methacholine, and thereby affecting the changes in all 3 mechanics parameters.

Our studies using human bronchial smooth muscle cells cultured *in vitro* show that HDM-induced morphologic change is attenuated in the presence of BHB and is at least partially a consequence of BHB-mediated inhibition of HDM protease activity. We posit that providing elevated levels of ketone bodies could provide benefit^65^ to patients with allergic asthma and may do so through a number of direct and indirect effects on lung physiology throughout the proximal airways and distal airspaces that modulate inhaled methacholine hyperresponsiveness. Although our studies reveal 1 mechanism by which ketone bodies may beneficially affect allergic asthma by attenuating HDM-induced effects on bronchial smooth muscle cells, the anti-inflammatory effects observed *in vitro* may also be evoked if higher concentrations of BHB are achieved *in vivo*, perhaps through pharmacologic instead of dietary means. Indeed, several studies of *in vivo* anti-inflammatory activities of therapeutic ketosis have been reported, with some directly relevant to lung inflammation.^18,34,35^ Intriguingly, it has been shown that ketogenic diet inhibits allergic airway inflammation elicited by the protease papain by decreasing type 2 innate lymphoid cell influx, activation, and accompanying cytokine production, although the mechanism described was related to decreased glucose availability rather than to the effects of ketone bodies *per se*, and methacholine responsiveness was not assessed.^66^

Elevating ketone bodies is safe both in animal models of disease and in human subjects.^22,23,40,67–73^ In our studies, feeding a ketogenic diet, adding 1,3-BD to chow, or supplementing with KE augmented circulating BHB levels, but not to remarkably high concentrations and not nearly to those used in our *in vitro* studies or in those of other investigators. A ketogenic diet contains sufficient protein, reduced amounts of carbohydrates, and an abundance of fat that serves as a substrate for ketone body formation.^74^ 1,3-BD is an ethanol dimer that is converted by the liver into BHB that can enter the circulation^15^ and has been incorporated into KEs. KEs are considered a dietary supplement and have shown benefits to elite athletes and in patients with chronic disease.^22,23,40,67,68^ As used in our studies, the KE (*R*)-1,3-BHB (*R*)-1,3-BD^23,67,68^ augments circulating BHB levels and was incorporated into mouse food at a concentration of 20% of weight (and approximately 20% of calories) to promote protracted consumption. KE supplementation elicited the most consistent and substantial effects to decrease methacholine hyperresponsiveness in each of the mouse models of allergic asthma, which we speculate is a consequence of its capacity to most markedly elevate circulating BHB levels. This KE supplementation strategy could be optimized as an approach to promote a state of “therapeutic ketosis” similar to that achieved through the feeding of a ketogenic diet or fasting, without any caloric deficit or the need for substantial lifestyle modification.

BHB has been reported to function as a class I histone deacetylase (HDAC) inhibitor^11,12,32^ and to induce β-hydroxybutyrylation of histone H3 lysines^75,76^ to influence gene expression. Histone modifications have been reported in the context of allergic asthma.^77,78^ β-Hydroxybutyrylation also posttranslationally modifies a multitude of additional cellular proteins with both known effects and heretofore unknown consequences.^79,80^ Although we speculated that hydroxybutyrylation of HDM proteins could account for the ability of BHB to inhibit HDM protease activity, inconsistencies of available reagents did not enable us to provide compelling evidence to support this hypothesis. As we have previously reported altered levels of expression of the smooth muscle–associated genes transgelin/SM-22 alpha (*Tagln*) and actin alpha-2/alpha-smooth muscle actin (*Acta2*) in the lungs of methacholine-hyperresponsive HFD-fed obese mice, which were decreased in obese mice consuming a KE supplement, bronchial smooth muscle cells *in vivo* may perhaps be targets of BHB-induced posttranslational modifications affecting gene expression or protein functions related to methacholine hyperresponsiveness. The effects of BHB on methacholine-induced intracellular signaling events in bronchial smooth muscle cells merit further investigation. Intriguingly, inflammasome activation^18,34–36^ and PAD4-regulated netosis^81^ are both inhibited by BHB, which decreases the regulated secretion of bioactive products, including IL-1β and NETs, respectively. It is possible that in addition to inhibiting bronchial smooth muscle contraction, BHB inhibits mucus secretion from methacholine-stimulated airway goblet cells, which could affect airflow and, therefore, the parameters measured in our flexiVent analysis.

There are several limitations to our findings. Namely, we used *in vitro* studies, cell lines, and preclinical mouse models instead of human subjects. The allergic asthma models used are not representative of the protracted nature of human asthma, and end points such as fibrotic remodeling were not assessed. Furthermore, whereas methacholine hyperresponsiveness is a relevant end point to assess in mice, it is not truly a surrogate for important human asthma–associated symptoms or morbidities such as exacerbation frequency, dyspnea, or impact on quality of life. Nevertheless, the effectiveness of therapeutic ketosis in decreasing the methacholine hyperresponsiveness associated with allergic asthma in several models implicates the potentially broad effectiveness of therapeutic ketosis in several asthma endotypes. Asthma “endotyping” is an approach in which clinical presentation and biomarkers are used to stratify patients and identify targets for disease intervention.^3,82^ We used HDM-driven preclinical allergic asthma models representative of T_H_2-dominated, eosinophilic, acute disease that is sensitive to corticosteroid treatment (alum/HDM and intranasal HDM sensitization),^52,83^ a HDM reexposure model representative of more protracted disease, and a model of mixed granulocytic, steroid-resistant, severe disease.^52^ Interestingly, in this model of severe allergic asthma, combined treatment with dietary KE and systemic corticosteroid significantly decreased methacholine hyperresponsiveness, particularly airway resistance, but also tissue damping and tissue elastance at the highest doses of methacholine. Add-on biologic therapy and corticosteroid administration is routinely used in the treatment of T_H_2^high^ asthma,^84^ and it would likely be used in novel non-T_H_2 severe asthma therapies as well. Although it is possibly only one of several approaches that may be useful, perhaps the combined dietary ketone supplementation plus corticosteroid treatment represents an optimal therapeutic strategy. How mice receiving dietary KE supplementation “reestablish” corticosteroid responsiveness remains unclear. As is already being tested,^85^ metabolic therapies for allergic asthma, including “therapeutic ketosis,” could provide potential complements or alternatives to other conventional approaches for the treatment of these patients.

## Supplementary Material

1

## Abbreviations used

AcAc: Acetoacetate
BALF: Bronchoalveolar lavage fluid
1,3-BD: 1,3-Butanediol
BHB: β-hydroxybutyrate
CFA: Complete Freund adjuvant
HBSMC: Human bronchial smooth muscle cell
HDM: House dust mite
KE: Ketone ester
NET: Neutrophil extracellular trap
PAD4: Polyvinylidene difluoride
PEEP: Positive end-expiratory pressure
Zrs: Respiratory system impedance
