# Supplementary material for: Ketone body augmentation decreases methacholine hyperresponsiveness in mouse models of allergic asthma
Source: J Allergy Clin Immunol Glob. 2022 Sep 7;1(4):282–98. doi: 10.1016/j.jacig.2022.08.001 (PMC9718535; doi:10.1016/j.jacig.2022.08.001)
Supplement: Fig E1 [file mmc1.pdf]

Supplement 1

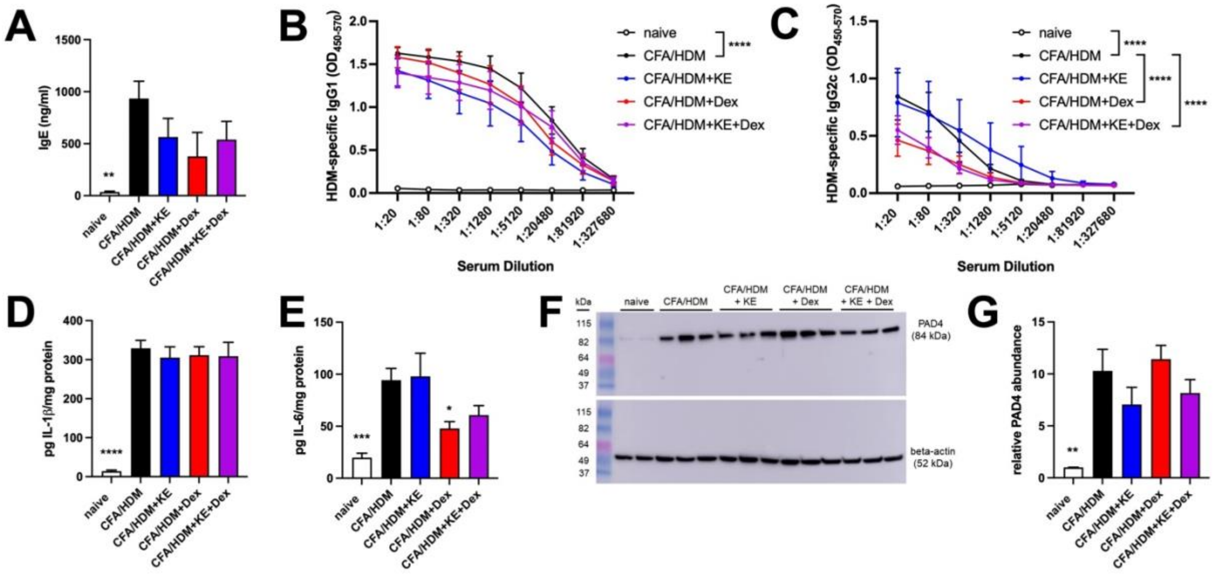

Supplement 1. Effects of ketone ester supplementation and corticosteroid treatment on serum immunoglobulins, lung tissue cytokines, and lung tissue PAD4 abundance in a model of antigen-driven mixed-granulocytic severe asthma. From mice trerated as described in Figure 7, total IgE **(A)**, HDM-specific IgG1 **(B)**, and HDM-specific IgG2c **(C)** were measured from serum. IL-1β **(D)**, IL-6 **(E)**, and PAD4 **(F** and **G)** were measured from lung tissue lysates. N=10 mice/group (A-E) or N=2 naïve and 3 experimental mice/group (F and G). * = p≤0.05, ** = p≤0.01, *** = p≤0.001, **** = p≤0.0001 compared to CFA/HDM (A, D, E, G) or indicated group (B, C).
